# TRIXcell+, a new long-term boar semen extender containing whey protein with higher preservation capacity and litter size

**Published:** 2014-02-28

**Authors:** B.M. van den Berg, J. Reesink, W. Reesink

**Affiliations:** 1 *Barex Biochemical Products, Enkhuizen, The Netherlands*; 2 *KI Mobiel, Dedemsvaart, The Netherlands*; 3 *Varkens KI Totaal, Ruinerwold, The Netherlands*

**Keywords:** Artificial insemination, Boar, Fertility, Semen preservation medium, Whey protein

## Abstract

It was the aim of the present study to test whey as protective protein for the sperm cell in the long-term boar semen preservation medium TRIXcell. Analyses of sperm cell motility using computer-assisted semen analysis (CASA) indicated that the whey protein Porex has a similar protective effect as bovine serum albumin (BSA) in maintaining viability of stored boar sperm. Boar sperm diluted in TRIXcell+ maintains commercially acceptable motility (>60%) for 10 days, while swine sperm diluted in the semen preservation medium Beltsville Thawing Solution (BTS) maintains commercially acceptable motility (>60%) for 3-5 days for most boars. To test the on-farm fertility performance of TRIXcell+ compared to BTS, inseminations were started on 35 commercial pig production farms in the summer of 2006. During the period of July 2006 until July 2012 for each farm and each calendar year the mean farrowing rate and litter size for semen diluted in TRIXcell+ and stored for 3-5 days was found higher than that of semen stored for 1-2 days in BTS. Based on data gained from a total of 583.749 sows inseminated through the years 2006-2012, the mean farrowing rate for semen diluted in TRIXcell+ and BTS was 90.4 ± 4.0 and 87.9 ± 3.6, respectively, which is not significantly different. Based on the same data, the mean total number of piglets born alive for semen diluted in TRIXcell+ and BTS was 14.2 ± 0.7 and 13.6 ± 0.6, respectively, which is significantly different. We conclude that whey protein can effectively be used in the long-term preservation medium TRIXcell resulting in a higher litter size.

## Introduction

The widespread use of artificial insemination (AI) in pig production has led to the development of highly specialized and professional AI centers that supply high quality diluted semen to their customers (Gerrits *et al.*, 2005; Feitsma, 2009). In addition to dilution of the semen, the use of semen preservation media is aimed at improving preservation capabilities by adding protective compounds such as bovine serum albumin (BSA), antioxidants and antibiotics (Johnson *et al.*, 2000; Levis, 2000; Gadea, 2003).

The worldwide most-used preservation medium for swine semen dilution is the Beltsville Thawing Solution (BTS) (Pursel and Johnson, 1975). This is a so-called short-term preservation medium which keeps the sperm viable for most boars when stored at 16-18 °C for 1-3 days. In most cases, insemination is done the day of production or the day after the production of the seminal dose. In contrast to BTS, a long-term preservation medium keeps the sperm viable for over 3 days, the number of days depending on the type of long-term preservation medium (Gadea, 2003).

Several new long-term preservation media have been introduced in recent years (Weitze, 1990; Gadea, 2003). These new preservation media have been tested using different in vitro methods (Dubé *et al.*, 2004; Vyt *et al.*, 2004) and by on-farm trials (Anil *et al.*, 2004; Haugan *et al.*, 2007). However, widespread use of long term preservation media is limited due to the price when compared to BTS (Waterhouse *et al.*, 2004; Haugan *et al.*, 2007).

Further, a lack of large-scale comparative application of different commercial preservation media from independent research institutes makes it difficult to compare and evaluate the real value of long-term preservation media. But in particular the past decade, there is a trend to replace BTS with long-term preservation media because the latter have practical advantages such as reduction of delivery days to customers and improved management of production and delivery of diluted semen (Kuster and Althouse, 1999; Haugan *et al.*, 2005).

A potential further improvement of boar semen preservation media lies in the replacement of bovine serum albumin (BSA), which is widely applied as the most commonly used protective protein in boar semen preservation media (Gadea, 2003). Since BSA is derived from cow’s blood, it would be better to have an alternative for application in preservation media, since cow’s blood may be related to the occurrence of bovine spongiform encephalopathy (Colchester and Colchester, 2006).

This paper describes the results of motility studies using computer-assisted semen analysis (CASA) and large-scale inseminations of a new preservation medium that is based on the long-term extender TRIXcell supplemented with whey protein to replace BSA.

## Materials and Methods

### Animals and semen collection

Semen was collected on a routine basis at the AI Stations of Varkens KI Service (Staphorst and Punthorst, The Netherlands) using a standardized protocol. Sexually mature boars, mostly between 1 and 3 years old of the breeds Duroc, Pietrain, York, Primeur, and Hampshire, were used to collect semen for research purpose and for distribution of the diluted semen to customers. Boars were housed in individual pens (± 9 sq. m.) in environmentally controlled farm buildings. They were given ad libitum access to water and were fed commercial diets according to the nutritional requirements for adult boars (Brown, 1994).

Semen was collected in the boar pens using the gloved hand technique (Hancock and Hovel, 1959) and was filtered through four layers of sterile gauze into a pre-warmed beaker to remove gel particles during collection. The semen was immediately diluted with approximately the same volume of appropriate preservation medium that was kept at 30 °C. To compare the use of BTS and TRIXcell+ in on-farm trials, care was taken to use ejaculates of same boars for both preservation media, to exclude a boar effect. The pre-diluted semen was then transferred to the laboratory in insulated beakers for further processing.

### Boar semen preservation media

The commercial semen preservation medium TRIXcell with BSA was purchased from IMV Technologies (L’Aigle, France). The TRIXcell semen preservation medium without BSA but with 0.1% whey protein (w/v) was named TRIXcell+. Both preservation media have a similar composition, but next to the protein difference, another difference is the composition of the antibiotics. We have used gentamycin (0.06 % w/v) and amoxycillin (0.06 % w/v).

The chemical composition of TRIXcell is proprietary. Beltsville thawing solution (BTS), and chemicals and antibiotics used to prepare the TRIXcell preservation media were purchased from Sinus Biochemistry and Electrophoresis (Heidelberg, Germany). The exact chemical composition of DiluPorc BTS from Sinus Biochemistry and Electrophoresis is unknown but the recipe for BTS is 37.0 g glucose, 1.25 g EDTA, 6.0 g sodium citrate, 1.25 g sodium bicarbonate and 0.75 g potassium chloride for 1 L of preservation medium as has been reported by Johnson *et al*. (2000).

Whey protein with brand name Porex was purchased from Phenolix (Enkhuizen, The Netherlands). Phenolix has stated that Porex has been produced from milk sourced from dairy cows that have been kept and managed according to the European Community Regulation 999/2001, which has been set in place to enforce the rules for the prevention, control and eradication of certain Transmissible Spongiform Encephalopathies (TSE). Ultra-pure water was used to prepare the preservation media.

The motility analyses as well as the on-farm inseminations were done using semen diluted in freshly prepared preservation media based on mixing the individual chemical components in the lab and solubilising the mixture at the day of semen collection.

### Comparative in vitro analysis of swine semen stored and diluted in BTS, TRIXcell, and TRIXcell+

Many experiments were carried out to test the motility of semen diluted in TRIXcell supplemented with Porex. For the illustrative motility experiment presented here, semen of 3 Pietrain boars that were known to give average quality sperm was collected and pooled, separated into 3 fractions and diluted using the appropriate preservation media to a concentration of approximately 30 × 10^6^ sperm cells per ml.

The analysis of sperm motility to compare the storage capability of BTS, TRIXcell, and TRIXcell+ was done using the Ultimate Sperm Analyzer from Hamilton Thorne (Beverly, MA, USA). The software settings recommended by Hamilton Thorne were used. Sperm cell samples were stored in 1 ml propylene tubes at 17 ± 1 °C and were only used once for analysis to prevent any effect of shaking the sedimented sperm or opening the tube. Motility determinations were done in triplicate.

For assessment of motility and progressive motility, 3.5 µL of the diluted semen was pipetted onto a pre-warmed LEJA slide with 4 counting chambers and a chamber depth of 20 µm, incubated for 10 min. at 37 ± 1 °C and then immediately analysed using the CASA system. At least 5 multiple microscope fields or 1000 sperm cells were analyzed within 2 min. after mounting the slides. LEJA counting chambers were purchased from NIFA Technologies (Leeuwarden, The Netherlands).

### Semen processing and insemination for large-scale on-farm insemination trials

The concentration and motility of all pre-diluted semen samples was determined immediately after arriving in the laboratory using the CASA system of Microptics (Barcelona, Spain). For assessment of motility, 3.5 µL of the diluted semen was pipetted onto a pre-warmed LEJA slide with 4 counting chambers and a chamber depth of 20 µm and immediatedly analysed using the CASA system. The pre-diluted semen was further diluted based on the CASA results with appropriate preservation medium to a concentration of approximately 25 × 10^6^ sperm cells per mL. The pre-diluted semen was packed in Easy-Pack bags from Veltkamp (Lochem, The Netherlands) at a volume of 100 mL.

The bags were airtight sealed with no air trapped inside and stored at 17 ± 1 °C. For semen diluted in BTS, collection and delivery of the diluted semen was primarily on Mondays and Wednesdays, while inseminations were done within 2 days after delivery. For semen diluted in long-term preservation medium, collection and delivery was primarily on Fridays, while inseminations were done on day 3, 4 or 5 after delivery. Transport of diluted semen to the farms was done in climatized boxes at a temperature of 17 ± 1 °C. At the farms, the bags were stored at 17 ± 1 °C until use. Large-scale on-farm trials of TRIXcell+ along with BTS was done on 35 commercial pig production farms in The Netherlands. The number of gilts and sows per farm ranged between 300 and 1500.

Gilts and sows that were inseminated were of different genetic background: TOPIGS 20, TOPIGS 50, HYPOR, Danbred, and PIC lines. Data on farrowing rate and litter size (total number of piglets born alive) as main parameters of fertility were collected on-site and stored in a sow management program. For practical reasons we have presented the number of piglets born alive instead of total number of piglets born, since mummified foetuses are not always counted by farmers.

### Statistical analysis

Excel Software (Microsoft, Redmond, WA, USA) and GraphPad Instat (GraphPad Software, San Diego, California, USA) were used for calculation of means, standard deviations, and significance of differences of the motility data. Means were considered significantly different with a p-value < 0.05. The fertility data of the on-farm trials were retrieved from the data management program from the farms and imported in Excel. Excel and GraphPad Instat were used for statistical analysis (two-way analysis of variance; ANOVA) of the insemination data. The data on the farrowing rate and total number of piglets born alive for both extenders were subjected to pair-wise comparison based on the Tukey method in conjunction with ANOVA to compare the differences. Means were considered significantly different with p < 0.05.

## Results

### Motility analysis of boar semen diluted and stored in BTS, TRIXcell, and TRIXcell+

Table 1 shows the results of a typical example of the analysis of the motility of sperm diluted and stored in BTS, TRIXcell, and TRIXcell+ during 10 days at 17 ± 1 °C. The percentage of motile sperm cells decreased with storage time for all three preservation media, but the percentage of motile sperm cells in the preservation media TRIXcell and TRIXcell+ remained higher when compared to BTS.

**Table 1:** Percentage (mean ± standard deviation) of motile and progressive motile sperm cells from day 0 to day 10 after collection and dilution of the sperm in the preservation media BTS, TRIXcell, and TRIXcell+ at a concentration of approximately 30 × 106 sperm cells per mL. The group p-value gives the result of comparison of means between BTS and TRIXcell+, as well as TRIXcell and TRIXcell+.

Day after collection	BTS	TRIXcell+	Group	BTS	TRIXcell+	Group
	Motility (%)	Motility (%)	p-value	Progressive motility (%)	Progressive motility (%)	p-value
Day 0	88.2 ± 4.0	89.0 ± 4.3	0.70 b	68.4 ± 3.3	70.2 ± 2.9	0.47 b
Day 2	77.2 ± 2.9	90.2 ± 3.5	0.03 a	58.3 ± 2.5	71.2 ± 2.8	0.02 a
Day 4	68.6 ± 2.5	90.7 ± 4.1	0.01 a	53.2 ± 3.0	68.3 ± 3.6	0.01 a
Day 6	43.5 ± 3.3	88.8 ± 3.5	<0.01^a^	25.7 ± 2.3	65.8 ± 2.7	<0.01 a
Day 8	22.2 ± 2.1	84.1 ± 4.2	<0.01 a	15.1 ± 1.9	66.7 ± 3.6	<0.01 a
Day 10	8.9 ± 2.7	75.2 ± 2.1	<0.01 a	3.4 ± 2.8	55.3 ± 3.3	<0.01 a

Day after collection	TRIXcell	TRIXcell+	Group	TRIXcell	TRIXcell+	Group
	Motility (%)	Motility (%)	p-value	Progressive motility (%)	Progressive motility (%)	p-value
Day 0	90.1 ± 2.5	89.0 ± 4.3	0.60 b	73.1 ± 3.1	70.2 ± 2.9	0.29 b
Day 2	91.1 ± 3.2	90.2 ± 3.5	0.63 b	72.9 ± 2.0	71.2 ± 2.8	0.38 b
Day 4	88.7 ± 1.9	90.7 ± 4.1	0.34 b	67.6 ± 4.1	68.3 ± 3.6	0.71 b
Day 6	86.4 ± 2.7	88.8 ± 3.5	0.29 b	63.2 ± 4.3	65.8 ± 2.7	0.32 b
Day 8	84.2 ± 2.5	84.1 ± 4.2	0.95 b	64.8 ± 2.3	66.7 ± 3.6	0.44 b
Day 10	78.6 ± 2.4	75.2 ± 2.1	0.23 b	58.2 ± 2.8	55.3 ± 3.3	0.24 b

a significantly different.

b not significantly different.

In the case of the preservation medium BTS the motility dropped below the commercial threshold of 60% at day 4, while both TRIXcell and TRIXcell+ had even at day 10 a motility percentage that indicated good quality for commercial application. The data show that there is no significant difference between the storage capacity of TRIXcell and TRIXcell+. Also in many other experiments (data not shown) TRIXcell and TRIXcell+ showed a similar storage capacity as indicated by motility.

### Inseminations with semen diluted in BTS and TRIXcell+

To confirm the indication of the motility studies, large-scale on-farm inseminations were started in July 2006.

A total of 35 swine production farms participated in using both BTS and TRIXcell+ diluted sperm. The number of inseminations using TRIXcell+ versus BTS varied per farm, while the number of gilts and sows per farm ranged between 300 and 1500.

Table 2 shows the fertility results of the large-scale inseminations separated per calendar year, as well as the total over 6 years.

**Table 2:** Annual and total fertility results (mean ± standard deviation) of inseminations done between July 1, 2006 and July 1, 2012 with semen diluted in TRIXcell+ and BTS.

Year	BTS	TRIXcell+	BTS	TRIXcell+	Group	BTS	TRIXcell+	Group
	N	N	FR	FR	p-value	TBA	TBA	p-value
2006	123892	3650	86.5 ± 3.4	90.2 ± 4.7	0.22 b	12.6 ± 0.7	13.4 ± 0.5	0.02^a^
2007	59625	20375	86.9 ± 3.3	90.0 ± 4.1	0.32 b	13.2 ± 0.9	13.8 ± 0.6	0.03^a^
2008	85320	25240	88.6 ± 3.2	89.7 ± 3.6	0.66 b	13.3 ± 0.4	13.9 ± 0.6	0.04^a^
2009	86408	15720	88.0 ± 4.0	90.2 ± 2.8	0.42 b	14.3 ± 0.3	14.9 ± 0.9	0.03^a^
2010	5520	64158	89.1 ± 3.7	91.0 ± 4.0	0.53 b	14.1 ± 0.4	14.4 ± 0.7	0.36 b
2011	6250	60015	87.4 ± 3.6	90.5 ± 3.7	0.33 b	13.7 ± 0.6	14.3 ± 0.6	0.02^a^
2012	5262	22314	88.5 ± 3.5	91.3 ± 3.0	0.40 b	13.8 ± 0.6	14.7 ± 0.5	0.02^a^
Total	372277	211472	87.9 ± 3.6	90.4 ± 4.0	0.25 b	13.6 ± 0.6	14.2 ± 0.7	0.03^a^

N = number of sows inseminated.

FR = farrowing rate.

TBA = total number of piglets born alive.

a significantly different.

b not significantly different.

The results show that for each calendar year both the farrowing rate and the total number of piglets born alive was higher for TRIXcell+ when compared to BTS.

The data also show that for a total of 583.749 sows inseminated through the years 2006-2012 the mean farrowing rate was 2.5% higher and the total number of piglets born alive was 0.6 higher for semen diluted in TRIXcell+ when compared to BTS. The higher farrowing rate is not statistically significant, whereas the 0.6 higher litter size is significant (P < 0.05).

## Discussion

Our study was started to find an alternative to BSA for application in semen preservation media. We focused our studies on whey protein since whey is an attractive alternative to BSA from cow’s blood. First, whey contains low amounts of BSA. Second, whey protein is not related to the occurrence of BSE and third, whey is much cheaper than BSA.

To test replacement of BSA by the whey product Porex we used various long-term preservation media such as TRIXcell, Androhep, Zorlesco and Modeno (Gadea, 2003).

However, for extensive on-farm testing we have chosen TRIXcell (earlier also named Tri-X-cell or X-cell), since various studies on the use of TRIXcell have been reported.

The results of these studies, in vitro studies (Waterhouse *et al.*, 2004; DE Ambrogi *et al.*, 2006; Estienne *et al.*, 2007; Lange-Consiglio *et al.*, 2013) as well as on-farm insemination trials (Kuster and Althouse, 1999; Haugan *et al.*, 2007), showed that TRIXcell is an efficient long-term preservation medium. Motility of boar sperm cells, both fresh and stored, depends on many factors such as the genetic makeup, health and age of the boar, and the season. But it was not the aim of the current paper to report on the variables involved. Thus, of the many experiments we have done, we have presented here only a typical example of a CASA analysis that serves to show the results of motility of sperm when diluted in a long-term preservation medium containing BSA and Porex, with BTS as standard preservation medium.

Our motility studies showed that Porex, when added to TRIXcell to make TRIXcell+, gave similar results with regards to storage capacity when compared to TRIXcell, which contained BSA. Our studies indicated that Porex can replace BSA in TRIXcell without any significant change in storage capability as determined by CASA.

Results of CASA do not provide definite proof of fertility, since there is no clear relationship between sperm cell motility and fertility. Several studies appeared on this subject, but with conflicting results (Liu *et al.*, 1991; Holt *et al.*, 1997; Gadea, 2005; Broekhuijse *et al.*, 2012).

Allthough motility results may serve as indication of storage capacity of preservation media, definite proof of performance with respect to fertility can only be gained from on-farm insemination trials. Therefore, we started on-farm insemination trials in the summer of 2006 aimed at studying the performance of TRIXcell+ along with BTS with respect to farrowing rate and number of piglets born alive as main parameters of fertility or success of AI.

During the six year period of insemination trials, all 35 farms that participated had each year a higher farrowing rate and a higher number of piglets born alive when TRIXcell+ was used instead of BTS. But the consistently higher farrowing rate was not significantly different between the two preservation media. The difference in number of piglets born alive, however, is significant.

TRIXcell+ showed an average increase of the number of piglets born alive by 0.6. Earlier studies indicated that TRIXcell had a similar performance with respect to farrowing rate and litter size as BTS (Haugan *et al.*, 2007).

Therefore, we conclude that it may be the protective effect of the whey protein Porex that causes the higher number of piglets born alive and that Porex is an effective additive in the long-term semen extender TRIXcell.
